# Inhibitor induced conformational changes in SARS-COV-2 papain-like protease

**DOI:** 10.1038/s41598-022-15181-y

**Published:** 2022-07-08

**Authors:** Glaucio Monteiro Ferreira, Thanigaimalai Pillaiyar, Mario Hiroyuki Hirata, Antti Poso, Thales Kronenberger

**Affiliations:** 1 Department of Clinical and Toxicological Analyses, School of Pharmaceutical Sciences, University of Sao Paulo, Av Prof Lineu Prestes 580, São Paulo, 05508-000 Brazil; 2 Institute of Pharmacy, Pharmaceutical/Medicinal Chemistry and Tübingen Center for Academic Drug Discovery, Eberhard Karls University Tübingen, Auf der Morgenstelle 8, 72076 Tübingen, Germany; 3 Department of Oncology and Pneumonology, Internal Medicine VIII, University Hospital Tübingen, Otfried-Müller-Straße 10, 72076 Tübingen, Germany; 4 School of Pharmacy, Faculty of Health Sciences, University of Eastern Finland, 70211 Kuopio, Finland; 5 Tübingen Center for Academic Drug Discovery and Development (TüCAD2), 72076 Tübingen, Germany

**Keywords:** Protein analysis, Protein structure predictions, Virtual drug screening

## Abstract

SARS-CoV-2’s papain-like protease (PL^pro^) interaction with ligands has recently been explored with a myriad of crystal structures. We used molecular dynamics (MD) simulations to study different PL^pro^-ligand complexes, their ligand-induced conformational changes, and interactions. We focused on inhibitors reported with known IC_50_ against PL^pro^, namely GRL-0617, XR8-89, PLP_Snyder530, and Sander’s recently published compound 7 (CPD7), and compared these trajectories against the apostructure (Apo), with a total of around 60 µs worth simulation data. We aimed to study the conformational changes using molecular dynamics simulations for the inhibitors in the PL^pro^. PCA analyses and the MSM models revealed distinct conformations of PL^pro^ in the absence/presence of ligands and proposed that BL2-loop contributes to the accessibility of these inhibitors. Further, bulkier substituents closer to Tyr268 and Gln269 could improve inhibition of SARS-CoV-2 PL^pro^ by occupying the region between BL2-groove and BL2-loop, but we also expand on the relevance of exploring multiple PL^pro^ sub-pockets to improve inhibition.

## Introduction

The recent severe acute respiratory syndrome coronavirus 2 (SARS-CoV-2)^1^ has been a worldwide concern since its first report in December 2019 (Wuhan, China). The disease caused by this new coronavirus was identified by the World Health Organization (WHO), in February 2020, as Coronavirus Disease 2019 (COVID-19). Later, the outbreak was declared a pandemic in March 2020, and, by January 2022, ~ 378 million cumulative cases were recorded globally, with ~ 5.67 million registered deaths^2^.

Researchers around the world have been working on developing preventive and therapeutic agents against SARS-CoV-2^3^. Initially, eight vaccines were approved for full use, while the other six vaccines got approval for limited use against COVID-19^4,5^. However, the emergence of SARS-CoV-2 variants led to increased transmission and resistance, which is associated with antibody escape from the virus spike epitopes. Currently, efficient clinical triage and supportive care are essential to contain severe COVID-19 patients^2^. Despite the remarkable results of vaccination campaigns and the potential new drugs, in some countries, patients with COVID-19 are still treated with repurposed drugs. These drugs’ effects are often controversial due to the adverse events or the lack of fully proven clinical verification of their therapeutic effects against this disease. Consequently, there is still a need for novel treatments, and the investigation of potential drug targets remains a cornerstone when designing novel antiviral drugs^6^.

Previous studies reported SARS-CoV-2’s proteases as a source of attractive drug targets for antiviral development^7,8^. The SARS-CoV-2 main protease (M^pro^) was the focus of studies due to the availability of crystal structures^9^ and various groups have already exploited it for the development of inhibitors^10^. Recently, PF-07321332, an inhibitor of the main protease (M^pro^), was an emergency use of authorization by the FDA against SARS-CoV-2 infection^6^.

On the other hand, the SARS-CoV-2 virus has a conserved Papain-like protease (PL^pro^) that is vital for viral replication^11^. PL^pro^ is responsible for the proteolytic processing of the product of open reading frame 1a (ORF1a) in the replicase gene of CoV-2, a large viral polyprotein containing non-structural proteins, which form the replicase complex^11,12^. PL^pro^ recognizes and cleaves the LXGG consensus sequence (Leu-X-Gly-Gly, X refers to unspecific amino acids) of viral and host proteins^13^. Evidence suggests that PL^pro^ can cleave the interferon-stimulated gene product-15 (ISG15) modifier from melanoma differentiation-associated protein 5 (MDA5) to escape immune surveillance The regulation of MDA5 by ISG15 is critical for detecting cytosolic viral RNA and initiating an innate immune response^14,15^. Therefore, inhibition of PL^pro^ activity is crucial to stop the viral replication and disrupt its role in immune response evasion halt^16^.

**Figure 1:**
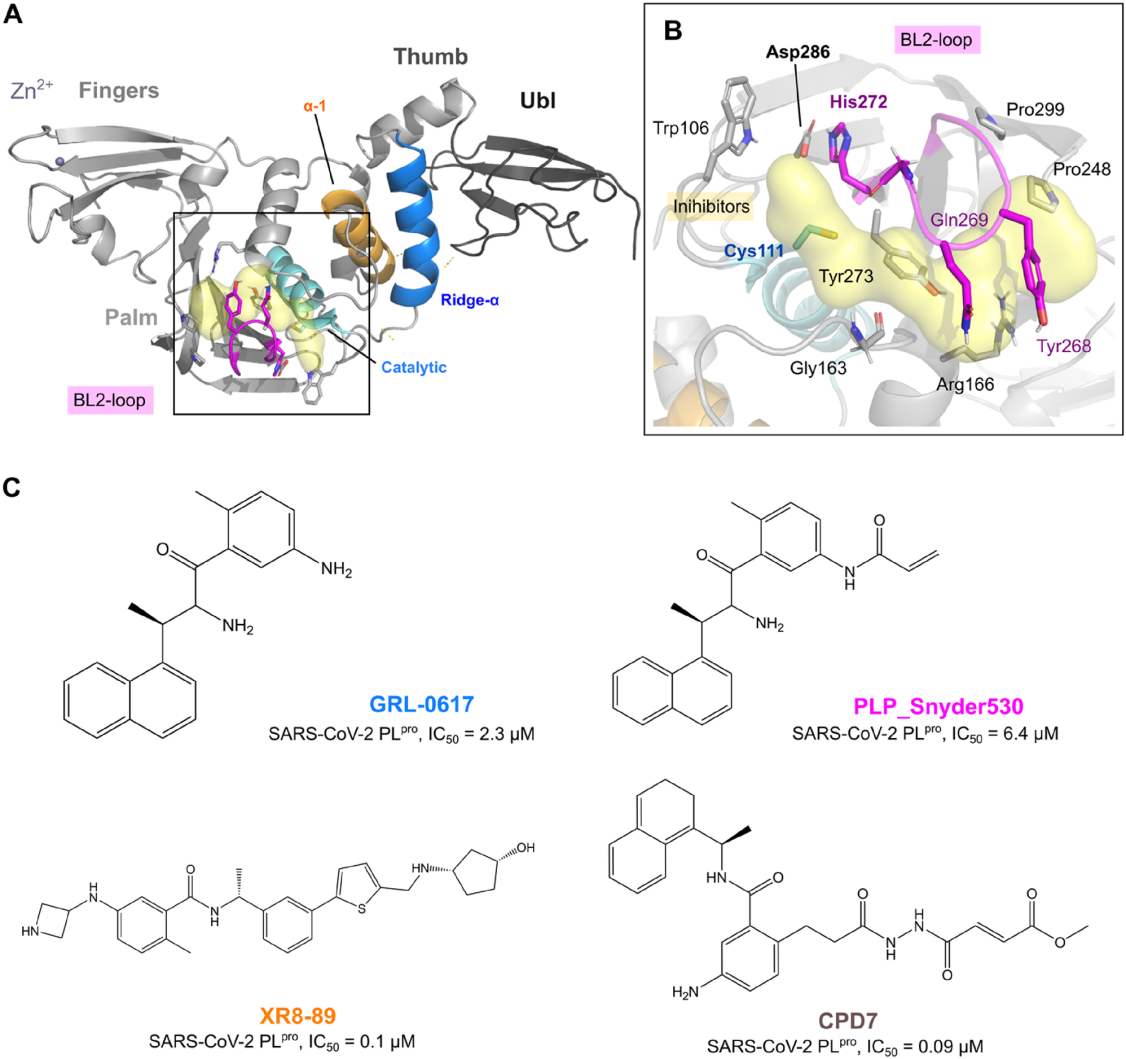
SARS-CoV-2 PL^pro^ structure overview, ligands from the protein data bank (PDB). (**A**) The tertiary structure of the SARS-CoV-2 PL^pro^ with domains UBL (1–60, in dark grey), Thumb (61–176, in grey), (**B**) Ligand binding pocket and catalytic triad (Cys111, His272, and Asp286^7^), Fingers (177–238), and Palm (239–315) and the BL2-loop (259–277, in magenta). (**C**) Two-dimensional representation of the ligands GRL-0617 (from PDB: 7jir), PLP_Snyder530 (from PDB: 7jiw), and XR8-89 (from PDB: 7lbr) and CPD7 (modelled as described in the methods section).

PL^pro^ consists of 315 residues and is biologically active as a monomer. It belongs to the ubiquitin-specific protease homolog family in humans^12,15,17^, and is topologically organized into four domains: Ubl (1–60), thumb (61–176), Ridge-α (76–90), fingers (177–238), and palm (239–315), as well as two sub-domains, BL2-groove (Pro248 and Pro299) and BL2-loop (259–277) (Fig. 1A)^13^. The Ubl and the finger region are connected by the so-called ridge helix (Pro77-Lys91), which was shown to be dispensable for MERS proteolytic activity^18^, but relevant to keeping SARS-CoV PL^pro^ solubility. The peptide bond cleavage in the active site is catalyzed by a conserved catalytic triad comprised of Cys111, His272, and Asp286 (Fig. 1B)^16^.

Yan et al*.*^19^ reported small molecule inhibitors targeting non-structural proteins (PL^pro^ and Mac1) and (M^pro^) of SARS-CoV-2. Another study reported potential antiviral effects of PL^pro^, namely GRL-0617, PLP_Snyder530 and XR8-89^16^. Further biochemical data on the PL^pro^ inhibition for GRL-0617 (SARS-CoV-2 PL^pro^, IC_50_ = 2.3 μM ± 0.5)^20^, PLP_Snyder530 (SARS-CoV-2 PL^pro^, IC_50_ = 6.4 μM ± 0.1)^13^ and for XR8-89 (SARS-CoV-2 PL^pro^, IC_50_ = 0.1 μM ± 0.03)^21^ indicates similar enzymatic inhibition, with XR8-89 being the most potent^21,22^ (Fig. 1C). PLP_Snyder530 has an acryloyl amino group instead of a single amine group on the benzamide moiety, in comparison with GRL-0617 and XR8-89, which slightly reduces this ligand’s potency^23^. On the other hand, ligands that have an N,N’-diacetylhydrazine group could access the BL2’s region to the active site and improve the activity against PL^pro^^24^. Recently, a set of GRL-0617 covalent derivatives were designed by introducing a peptidomimetic linker and reactive electrophilic “warheads”, with compound 7 (CPD7) being the most potent (IC_50_ = 0.094 µM^24^, on the proteolytic assay) and were also selected to be simulated in our study.

Interestingly, crystal structures of these inhibitors within the PL^pro^ binding pocket display ligand interactions with the BL2 groove (amino acids Pro248 and Pro299), the flexible BL2 loop (259–277)^17^ and the site is distal from the active site’s cysteine^21^, suggesting ligand-induced conformational changes manly in BL2’ region and could increase the activity against PL^pro^.

Previous, simulations of PL^pro^’s from different viruses showed that the overall dynamics of ligand-free PL^pro^ were similar, with comparable flexibility in key regions, such as the BL2 loop, zinc-binding region and Ubl. However, the availability of diverse ligands at that time would be insufficient for a more detailed description of ligand-induced conformational changes.

Here, we aimed to expand on the inhibitor-induced conformational changes, using longer molecular dynamics simulations applied on crystals/models of relevant PL^pro^ ligands. We believe our results can help future drug screening/optimization studies that would employ modelling.

## Results

We report microsecond MD simulations of the SARS-CoV-2 PL^pro^. Each analyzed system was subjected to at least 10 μs simulation time, spread among at least five independent replicas. We compared reported inhibitors, namely GRL-0617, PLP_Snyder530, XR8-89 and CPD7, both covalently bond and free^15,22,24^, against the apostructure to study the changes induced by the ligand binding, with a total of around 60 µs worth simulation data.

### Conformational changes in domains far from PL^pro^’s active site influence the BL2-loop and BL2-groove

**Figure 2:**
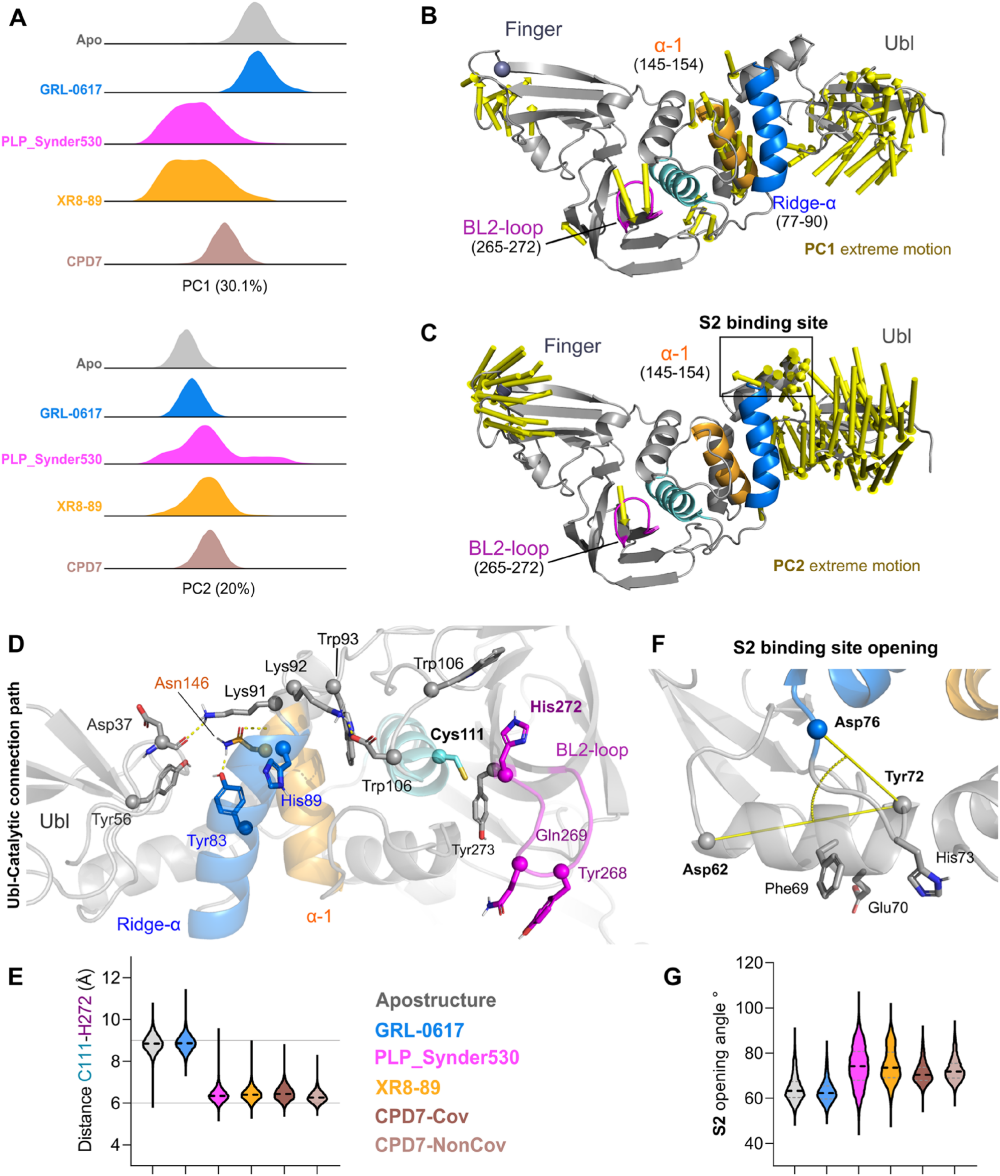
SARS-CoV-2 PL^pro^ principal component analysis. (**A**) Distributions over the two significant PCs (PC1 and PC2) separated for each simulated system apostructure (PDB: 7lbr) is highlighted in grey; GRL-0617 bound structures (GLR067-7JIR) in sky-blue; PLP_Synder530 bound structures (PLP_Synder530 - 7JIW) in magenta, XR8-89 bound structures (XR8-89 - 7LBR) in orange and CPD7 bound structures (CPD7) in dark brown. (**B**) Extreme motions from the PC1 displayed over the PL^pro^ tertiary structure represented by arrows. (**C**) Extreme motions from the PC2 displayed over the PL^pro^ tertiary structure represented by arrows. (**D**) Ubl-Catalytic connection path, with relevant residues highlighted as sticks (**E**) Distance values between C111 to H272 along with the simulations depicting the full extension of the inhibitor binding site opening. (**F**) S2 binding site opening representation, and (**G**) S2 vector angle analysis describing the opening/closing of this region. For E and G, ligands are coloured according to figure legend.

Principal Component Analyses (PCA) of all the concatenated trajectories of all simulations pooled together captured into PCs significant moments of PL^pro^. The first two components are accounting for PC1 30.8% and PC2 20.7% (all combined 51.5%, Fig. 2A) of the motion variability. The differences in the simulated systems can be explained by PC1/PC2’s distribution (Fig. 2A) as it separates apostructure and GRL-0617, each showing a single cluster, from the PLP_Snyder530, XR8-89 and CPD7, whose clusters are more dispersed. Meanwhile, PC2 distribution (Fig. 2A) of covariance separates a set of PLP_Snyder530 from the rest of the analyzed conformations.

PC1 extreme motion displays large mobility of the Ubl domain regarding the ridge helix, as well as an opening-closing movement of the BL2 loop (Fig. 2D). These two large conformational changes seem to be connected by the ridge (Pro77-Lys91) and thumb-helix (residues Ala145-Cys155) movements. Meanwhile, PC2 is much less connected to the active site conformation, describing the Ubl torsion towards the ridge-helix coordinated with the opening of the Zn-finger (Fig. 2C). Further, we observed motions in S2 binding site in PC2, with a smaller contribution in the PC1 (as observed by the arrows Fig. 2B,C).

The connection between Ubl and the “ridge” helices, as observed in crystal structures, is mediated by the interaction between Tyr56 and Tyr83 sidechains (Fig. 2D). However, upon simulation, there is a loss of this interaction, which allows the shift in Ubl conformation. Tyr83 side chain interacts with Tyr72 (water-mediated hydrogen bond, ~ 30%, Table S1) and is further stabilized by a hydrogen bond with Asn146 from the thumb helix (for ~ 65% of the analyzed simulation time). The conformational changes in the Ubl free the ridge to display more interactions with the catalytic domain (Supporting information, Table S1). The second point of contact between Ubl-Ridge is the Lys91 residue, which originally in crystal structures establishes a salt bridge with Asp37. However, our simulations show a more intermittent behaviour with interaction frequencies ranging from ~ 21 to 37%. Lys91 and Tyr83 interaction pattern changes allow the large movement of the Ubl domain, which frees the S2 binding pocket region to display conformational changes, as discussed below.

Overall, this ridge helix movement propagates towards the BL2 by a series of salt-bridges and polar contacts promoted by the Lys91-Trp93 motif (Fig. 2D). In this context, the Trp93’s sidechain works as a hub by interacting with the His89 (ridge helix) and the Asp108 (active site helix). This motif conformation is supported by the change from Lys92-Asp108 to His89-Asp108 interaction, which stabilizes the catalytic helix in an open conformation.

Overall, different conformations of the oxyanion region were observed for each inhibitor, for instance, the apostructure simulation and GRL-0617 display a large open caveat (as depicted by the distances between Cys111 and His272 C-alpha, Fig. 2D), where the His272 is stabilized by a water-mediated interaction with the Trp106 sidechain 39–41% of the analyzed simulation time. Interestingly, this interaction is absent in all the other simulations.

Patchett et al.^25^, discuss that the oxyanion hole may exist in various active and inactive conformations, based on the Trp106 position, substrate-binding status and step of the reaction. We monitored this amplitude variation by the changes in the distance between the Cα from Cys111 and His272 (Fig. 2E), which seems to be unoccupied in the GRL-0617 and apostructure simulations, where it is more constricted or occupied in the other studied systems.

The perpendicular helix to the ridge is known to host mutations on variants of concern of the virus (F69S, E70K and H73G). This region is proposed to constitute the substrate-binding site 2^25^. This site is relevant for the binding of multi-Ub substrates, working as a complementary binding site to the primary S1^25^. Considering the concerted movement observed in the PC2, we chose to investigate the variation of the opening angle in our trajectories (Fig. 2F,G). We observe a large angle range variation for some inhibitors, such as the PLP_Synder530, which is consistent with the PC2 expansive distribution for this compound. However, the implications of specific differences between the apostructure (with this region more compact) and the inhibited states (displaying a larger, more flexible S2 region) remain to be investigated.

The work of Patchett et al. has experimentally assessed S2 mutations on an in vitro level^25^, showing that the triple mutant remains active for mono-Ub substrates, but could not cleave K48-linked multi-Ub. Lastly, our PC2 extreme motion shows a large conformational change of the Zn-finger domain, moving from an extended open position, observed in the initial states, towards a more closed one.

**Figure 3:**
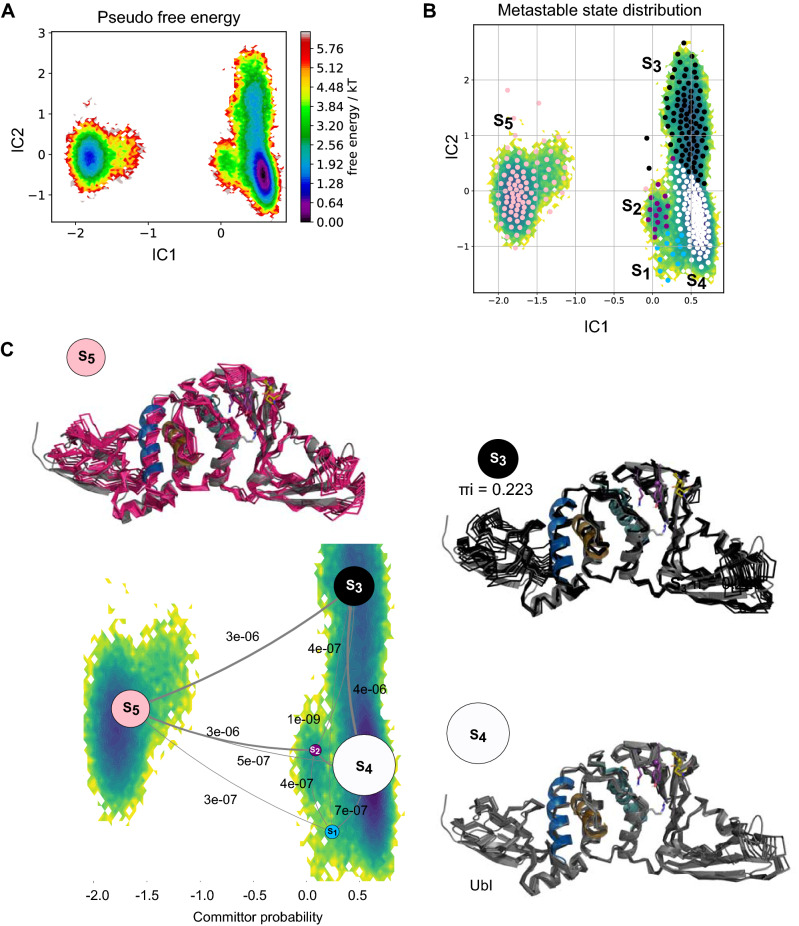
Metastable states of PL^pro^ in the presence and absence of the ligands as revealed by Markov state modelling. (**A**) Pseudo free energy map of distributions along with time-lagged independent components (ICs) 1 and 2. (**B**) Separation of five metastable states (S_1_–S_5_) by PCCA++ analysis. (**C**) Committor probability of the most representative metastable states. Each metastable state (S) is illustrated with ten representative structures (coloured ribbons), superimposed to a transparent cartoon with the original crystal structure. Equilibrium probability (πi) of distributions for each state is indicated below the conformations together with circles with an area representing the changes induced by the systems, values near to 1.0 more probable than values close to 0.

Based on these large changes, we hypothesized that different inhibitors would stabilize different protein conformations and, therefore, we aimed to use Markov State Models to identify these metastable states and their transition probabilities. MSM was used to identify possible metastases trough using the backbone torsions of whole protein domains (Ubl, Thumb, Triad, Palm, Finger, BL2-groove, and loop) in the presence of the ligands and Apo altogether. MSM consists of a master equation that can explain the probability transition between a set of conformational microstates, therefore describing the behavior of a system at long-time scales. MSM analysis revealed five metastable states (S_1_–S_5_; Fig. 3A,B), with three representative metastable (S_4_, πi = 0.529; Fig. 3C) for the Apo, GRL-0617, PLP_Snyder530, XR8-89 and CPD7 complex (Supplementary Fig. S1, displays the three most probable metastable states in comparison to the initially simulated conformations). Additionally, we observed an increase of flexibility in the finger and Ubl region among metastases S_3_ (πi = 0.223; Fig. 3C) and S_5_ (πi = 0.119; Fig. 3C), which can contribute to Ubl adoption and Zn-finger stabilization. We also analyzed the individual BL2-loop opening movement, by the means of the dihedral angle variation along with the simulations.

**Figure 4:**
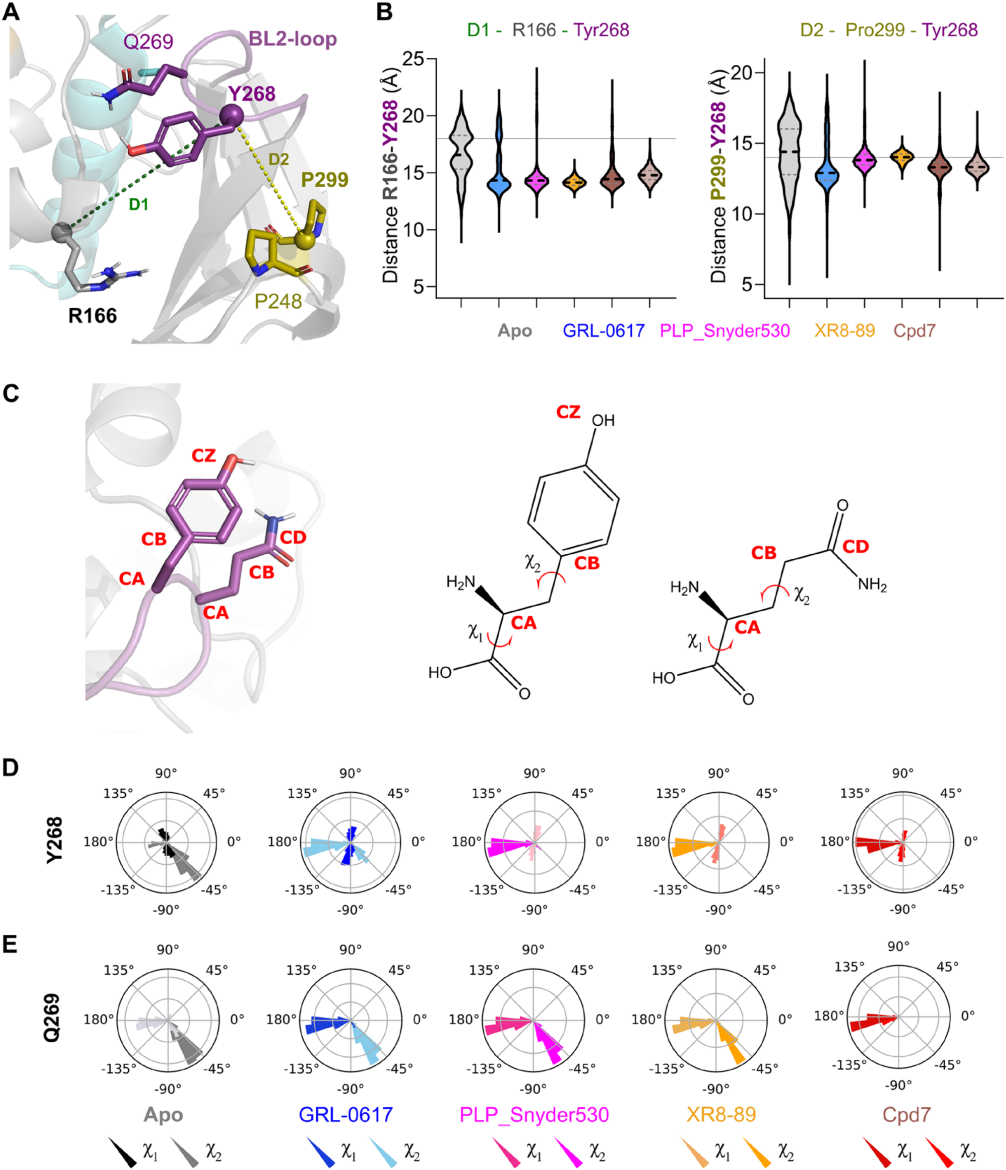
Dihedral angle distribution analysis for the residues BL2 loop (χ_1_ (CA to CB) and χ_2_ (CB to CD/CZ) angles conformation). (**A**) 3D structure representation of the distance between R166 (in grey), P299 (in sand) and Y268 (in magenta). (**B**) Distance between R166 to Y268 in absence and presence of the ligands and distance between Pro299 to Tyr268 in absence and presence of the ligands. (**C**) 2D and 3D representation of the χ angles in Y268 and Gln269 from apostructure and in presence of the ligands. For each graphic in (**D**,**E**) Dark coloured angles (black, dark blue, pink, dark orange and red) represents the angle χ_1_, which is the movement of Tyr289 and Gln269, while the light coloured bars represents the χ_2_, which is the angle of Tyr289 and Gln269. Graphics are coloured according to Apostructure by grey, GRL-0617 sky blue, PLP_Snyder530, magenta, XR8-89, orange and CPD7 covalently bound, red.

Originally in the crystal apostructures, the BL2-loop displays a majority of open conformation (illustrated in Fig. 4A) while it closes in presence of ligands. We then used the distances between the Cα of Tyr268 and two stable points Arg166 (Fig. 4A,B) and Pro299 (Fig. 4A,B) to describe the BL2-loop opening/closing movement. We observed that in presence of a ligand these distances are smaller when compared to apostructure, which has on average larger values and broader distributions. Meanwhile, the χ angles were analyzed to observe the movements of the residues localized in the BL2-loop site (Fig. 4C, for an illustration of the selection of the angle). We noticed that cooperative movements in the BL2-loop happened and the major difference was in Y268 from apostructure than in presence of the ligand (Fig. 4D,E).

Individually, the residues from BL2-loop showed differences among χ angle conformation to Tyr268 in absence of the ligand (Apo: − 45° to 90°) and the presence of the GRL-0617 (Fig. 4C; 45°–180°). At the same time, in presence of PLP_Snyder530, XR8-89 and CPD7 kept the χ angles between 90° and 180° (Fig. 4C). In contrast, the Gln269 to Apo and inhibitors (GRL-0617, PLP_Snyder530, XR8-89) presented two angles’ conformation (Fig. 4D; − 45° to 180°) while CPD7 showed one most representative angle at 180° (Fig. 4D).

Our geometric calculations contribute to describing a more diverse conformational landscape for the PL^pro^ inhibition. Unfortunately, very few geometric parameters seem to distinguish between the specific inhibitors, suggesting that for the main catalytic site a narrow conformational landscape is available. This prompted us to further study the protein–ligand interactions.

### Protein–ligand interaction by PL^pro^ inhibitors

**Figure 5:**
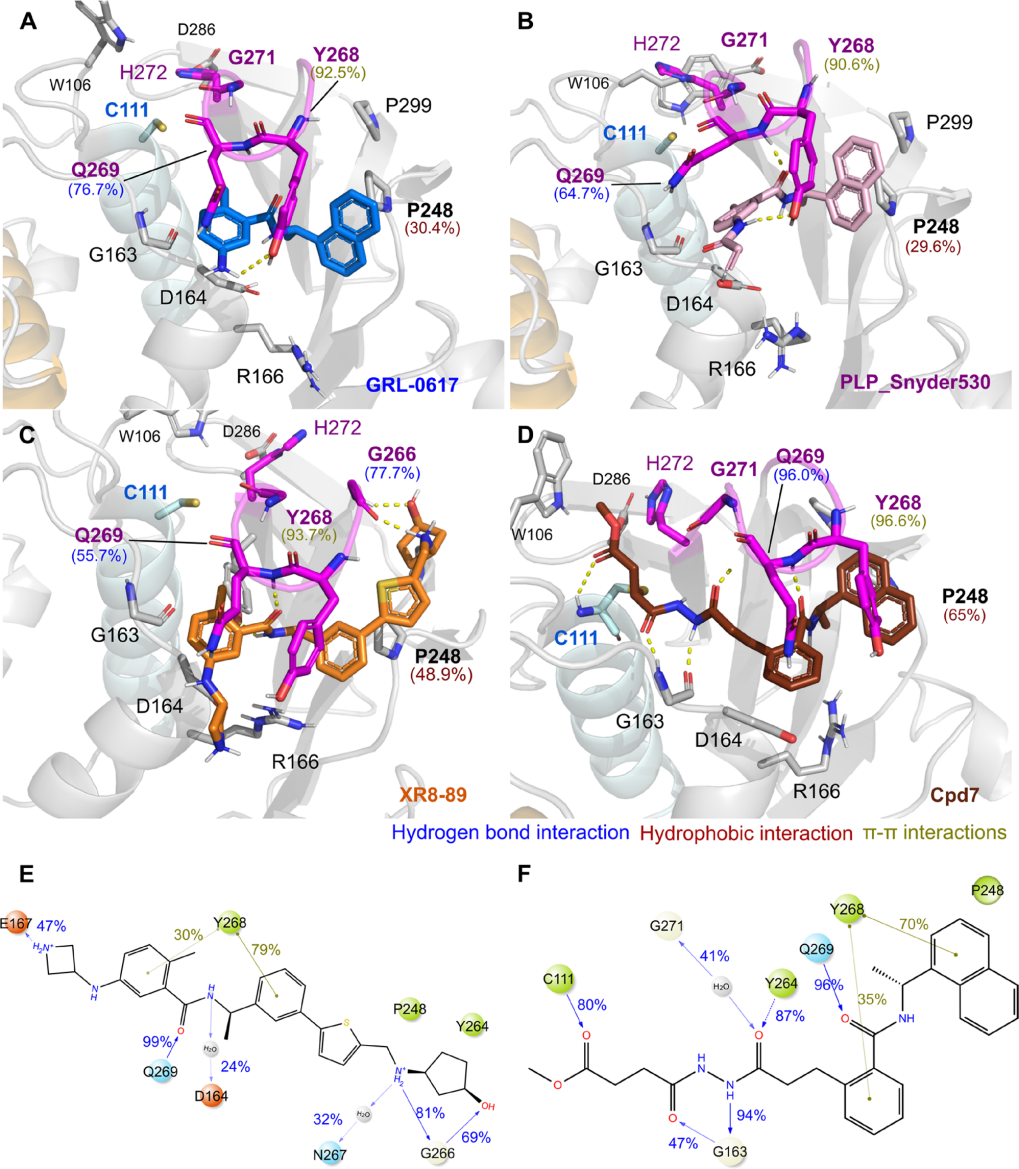
Overall interactions of the SARS-CoV-2 PL^pro^ with inhibitors. Snapshot frames of the simulations displaying relevant interactions between the PL^pro^ in the presence of the ligands GRL-0617 (**A**, depicted in sky blue); PLP_Snyder530 (**B**, magenta), XR8-89 (**C**, orange) and CPD7 (**D**, dark brown). 2D structures representations of XR8-89 (**E**) and CPD7 (**F**) and frequencies of interactions. PL^pro^ residues are colored according to the types of atoms in the interacting amino acid residues with the protein carbon, light grey; nitrogen, blue; oxygen, red.

PL^pro^’s catalytic site is far from the inhibitor binding pocket, which lies on the surface between the BL2 region (Fig. 5A–D). The most relevant amino acids stabilizing the ligand binding are Tyr268 and Gln269 (BL2-loop), Pro248 (BL2-grove) and Gly271 localized in Gly–Gly recognition site (substrate-binding sites)^13,18^.

All the ligands have a common benzamide moiety that establishes stable hydrogen bonding and π–π interactions with Tyr268 (H-bond; GRL-0617 ≤ 61%, PLP_Snyder530 ≤ 82%, XR8-89 ≤ 55% and CPD7 ≤ 70% and π–π GRL-0617 ≤ 92%, PLP_Snyder530 ≤ 90%, XR8-89 ≤ 93% and CPD7 ≤ 96%, Fig. 5A–D and Table S2). Another relevant interaction observed was the hydrogen bond with Gln269’s side chain, in the BL2-loop (GRL-0617 ≤ 76%, PLP_Snyder530 ≤ 64%, XR8-89 ≤ 55% and CPD7 ≤ 96%, Fig. 5A–D and Table S2). Hence, GRL-0617’s benzamide group displays a less frequent interaction with the sidechain of Arg166 (< 21%; Fig. 5A–D and Table S2). We also observed that naphthalene rings of GRL-0617 and PLP_Snyder530 occupy the hydrophobic pocket P3, displaying contacts with Pro248 in BL2-groove (GRL-0617 ≤ 31%, PLP_Snyder530 ≤ 29%, Fig. 5A–D and Table S2). This hydrophobic contact is more relevant for the XR8-89 and CPD7, which have a 2-phenylthiophene and naphthalene groups respectively (Pro248 hydrophobic frequency ≤ 49% and 65%, Fig. 5C–F and Table S2).

Additionally, GRL-0617 (< 52% and 3.9 Å; Fig. 5A and Table S2) and XR8-89’s (< 78% and 1.3 Å; Fig. 5C) have an extra amine group that formed a stable hydrogen bond interactions with this residue. However, CPD7’s N–N-hydrazone group interacts with Gly163 (~ 94%; Fig. 5D and Table S2) and other ligands do not interact with this residue.

We also observed, that Asp164 does not stably interact with the ligands, as originally suggested in the literature^22^, displaying seldom water-mediated interactions. In our simulations, Asp164 displays preferrable hydrogen bonds with Thr168, Glu167, or Arg166 (Table S3), depending on the bound ligand.

The Asp164-Thr168 interaction is stable with GRL-0617 (~ 95%), PLP_Snyder530 (~ 91%) and XR8-89 (≤ 96%, Table S3), while Asp164-Glu167 (GRL-0617 ≤ 44%, PLP_Snyder530 ≤ 43% and XR8-89 ≤ 45%, ; Table S3). Specifically, Asp164-Arg166 occurs intermittently for GRL-0617 (~ 20%), PLP_Snyder530 and XR8-89 (~ 40%, Table S3), in contrast to CPD7 simulations (≤ 81%, Table S3).

We also display a 2D representation of XR8-89 and CPD7 (Fig. 5E,F) to illustrate that, despite occupying a core common pocket, each of these inhibitor’s substitutions occupies unique sub-pockets which could be used in conjunction for future drug design. Both compounds XR8-89 (Fig. 5E) and CPD7 (Fig. 5F) presented the most stable interactions, interacting with important residues, such as Y268 (79%/70%) and Q269 (99%/96%). XR8-89 complementary interaction with Gly266 (69%)/G271 (41%) localized in Gly–Gly recognition contrasts with CPD7 interaction with Gly163 (94%), that responsible to kept the BL2-loop available for interaction.

## Discussion

According to our results, the conformational clusters (identified by the PCA) are distinct in PL^pro^ bound to inhibitors (GRL-0617, PLP_Snyder530, XR8-89 and CPD7) in comparison to apostructure simulations. Simulations with ligand-bound systems also show increased flexibility around the active site from PL^pro^ as seen by our increased RMSF variation (Supplementary Fig. S4), mainly in Finger, Palm and Ubl region. This was previously reported in shorter simulations with the apostructure^13^, with large Ubl conformational changes, and the range of mobility is known from comparing substrate-bound crystals with apostructures^21,22^, which shows different Finger conformations.

### PCA and MSM suggest the convergence to a set of relevant states for inhibited PL^pro^

The MSM states inferred larger conformational changes in regions, initially observed in the PCA, such as the Zn-Finger and the Ubl (Fig. 3), showing an overall opening of the PL^pro^. Interestingly, ubiquitin substrate (Ubl region) binds in an open state, by sitting on the Palm and the zinc-binding in the Finger region, which suggested that MSM states inferred the substrate-binding conformations^15,23^. These regions are located relatively far from the catalytic site^14^ and the classical inhibitor binding sites.

Previous apostructure simulations from Bosken et al. observed a partial rotation of Ubl relative position to the “ridge” helix (Asp62–His73) position^26^. A full description of the Ubl domain’s function remains unknown, however, some studies have shown MERS and CoV-1 PL^pro^ mutants with the deletion of the Ubl domain (ΔUbl) remain proteolytic active^18^.

Our results suggest that the conformational changes in the Ubl free the ridge to display more interactions with the catalytic domain, by the loss of the Tyr83-Tyr56 hydrogen bond, in exchange for Tyr83-Asn146. Interestingly, in MERS-CoV, the Tyr83 is substituted by Phe83 (Supplementary Fig. S3), which would not display hydrogen bond connections between Ubl and the rest of the protein.

Also, the MERS-CoV ridge helix is longer (from Thr78 until Trp93) displaying rigid characteristics, whereas the equivalent region on SARS-CoV-2 is more of a loop. We observed that this loop connects the ridge-α and catalytic helices and that its flexibility is relevant to propagating the movement towards the BL2-loop. Clasman et al*.* (2017) have also shown that SARS-CoV-1 PL^pro^-ΔUbl2 kept its DUB and protease activity, however displaying instability during expression and purification^18^. The specific role of Ubl in the SARS-CoV-2 PL^pro^ remains to be experimentally assessed, however, we propose based on our models that Ubl could play a role in maintaining the conformation of the Ub binding site S2. On the other hand, we noticed that change with Gly92 in MERS-CoV (Supplementary Fig. S3) and Lys92 in SARS-CoV-2 (Supplementary Fig. S3). We suggested that may be presented differences in the catalytic helix stabilization while the impact in PL^pro^ conformation.

We observed in the MSM analysis the metastable S_4_ as the most probable inhibited state (πi = 0.529; Fig. 3C and Supplementary Fig. S1)^27^, but all states display a range of conformations for the Zn-finger, from open to closed. Interestingly, crystal structures of PL^pro^ binding with ISG15-CTD substrate (PDBs 6XA9 and 6BI8, Supplementary Fig. S5, with the S_3_ state superimposed), therefore the catalytic competent state, display a range of Zn-finger conformations^15,22^.

The pivotal finding in the MSM analysis is the connection between the opening/closing of the finger and palm domains with the conformation of the BL2-loop, as observed from the conformational changes among the structures in S_3_ and S_5_. Further, Zn-finger and Ubl region in S_4_ seems stable, while they are not in the metastases S_3_ and S_5_. We suggested that states S_3_ and S_5_ represent the microstates before the stabilization. Finally, the S_4_ metastable state (πi = 0.529/52.9%) is the most probable and, based on its BL2-conformation (Supporting information Fig. S1), is proposed as a representative for a converged inhibited state.

One main limitation of our work is our focus on inhibitor development, as observed by the choice of our simulated systems. This leads us to conclude that with the currently available inhibited structures few conformational changes direct influences the inhibitor binding pocket, with domains far from this pocket potentially contributing to describing the inhibited states. Further simulations with different substrates and long apostructures simulations are in the scope of future research.

### The movement between BL2-groove and BL2-loop is key for the binding of the ligand in the PL^pro^ site

Conformational changes in the BL2-groove to BL2-loop are shown to regulate the binding of the PL^pro^ substrates^22^, with local mutations impacting its flexibility and activity^15,28^. We suggest a concerted mechanism between BL2-groove and loop to adopt the inhibited conformation. Shen et al.^22^ showed the cooperative binding in the BL2 regions by controlling the flexibility in BL2-loop through Tyr268 and Gln269. Also, the accessibility of the PL^pro^ active site is controlled by the BL2-loop^14^, which works as a gate through an unusual beta-turn formed by Tyr268 and Gln269^22,29^ (Fig. 4). Our apostructure trajectories could recapitulate this flexibility, with the BL2 loop displaying a large movement amplitude from “open” (large distances between the center of mass of the loop and the reference points) to “close” (smaller distances). Simulations with ligands GRL-0617 and PLP_Synder530 (least potent in terms of IC_50_) also visit these “open” conformations at some point during simulations. However, less frequently than the apostructure counterpart. Lastly, the most potent ligands, XR8-89 and Cpd7 stabilize the closed conformation of the BL2 loop along the entire analysed trajectory.

This BL2 loop structural diversity is only observed in the S_5_ (πi = 0.199), while states S_3_/S_4_ (in total πi = 0.752) characterize together a BL2 loop closed conformation, suggesting that in presence of inhibitors this is the most likely conformation (Supporting information, Fig. S1). Interestingly, the analysis of the BL2-loop angle variation by residues (Fig. 4 and Supplementary Fig. S2) showed of Tyr268 and Gln269 side-chains are observed inwards and outwards in apostructure, while only inwards the presence of the ligands^12,17^. In this way, the ligands would stabilize an inhibited state by interacting in the pocket between the BL2-groove and loop^17,22,30^, establishing relevant interactions with Tyr268, Gln269 (BL2-loop) and Gly266 (substrate binding site). These interactions were previously observed in the PL^pro^ crystal structures (PDB: 7lbr^22^, 7jir^17^ and 7jiw^22^) and, specifically interacting with the Tyr268 side-chain was shown to be essential for the inhibition^12,16,31^.

The benzamide of GRL-0617 establishes hydrogen bond interactions with Gln269 and the side chain of Asp164 in PL^pro^, thereby closing the BL2 loop^32,33^. Studies suggested that the binding of inhibitors could induce the BL2 loop closing, mediated by Tyr268 and Glu269 χ angles^17^. Further, the access by substrate or inhibitors in the PL^pro^ catalytic site is controlled by the BL2-loop^21^.

Lastly, the work from Gosh et al.^34^, suggests that the lack of potency by PL^pro^ inhibitors is the development being restricted to the substrate-binding site, where the large induced-fit mechanism should be accounted^22^ for. We proposed that these newly identified states can be combined to better represent the druggability landscape, especially where the BL2-loop reorganization is concerned. We highlight, due to the nature of the pocket, that a closed state is more druggable than an open state from the point of view of standard metrics^17,22^.

Our unsupervised all-atom simulations supported the stabilization of ligand binding by interactions with Tyr268 and Gln269 in the closed BL2 loop conformation. The work by Sohraby et al*.*^23^ also showed, using supervised MD simulations, the relevance of the BL2 opening in the ligand unbinding, due to the lost interactions with Gln269 and Tyr268 sidechains. They hypothesized that reducing the direct interactions with the BL2 loop and, complementarily, maximizing interactions with the inner parts of the binding pocket would improve ligand affinity. In agreement, we suggest inserting substituents bulkier compounds binding among BL2-groove and loop can help improve the binding affinity, but we also expand on the relevance of exploring multiple PL^pro^ sub-pockets to improve inhibition.

## Conclusions

PCA analyses suggest that PL^pro^ apostructure and GRL-0617 display similar conformational changes when compared to PLP_Synder530, XR8-89 and CPD7, which were further confirmed by geometric analyses of S2 site opening (more closed in the two first systems) and Opening of the inhibitor binding pocket (more accessible in apostructure and GRL-0617 simulations).

Concerning the movement between domains, the metastases generated by the MSM model suggest that the Finger and Ubl domain movements influence the accessibility of the inhibitors by the BL2-loop. Our simulations recapitulated the known observation that the BL2-loop open and closed conformation is key for keeping the stability of the inhibitor binding site.

Our simulations showed that large aromatic rings in the main core most frequently established interactions with Tyr268^31^. The Tyr268 and Gln269 residues, which are in BL2-loop^22,29^, are considered the most relevant in controlling host and viral protein binding substrates, which supports their role in inhibitor development. Further, observations from XR8-89 and CPD7 simulations illustrate that despite occupying a core common pocket, each of these inhibitor’s substitutions occupies unique sub-pockets adjacent to the BL2-loop, which could be used in conjunction for future drug design. Specifically, Pro248 from BL2-groove keeps the backbone of Gly266 available for potential hydrogen bonds with the inhibitors^21^.

## Experimental methods

### Modelling and structure preparation

The SARS-CoV-2 PL^pro^ apostructure (starting from PDB: 7LBR, Resolution: 2.20 Å and removing the ligands) and structures with ligands interacting with GRL-0617 (PDB: 7JIR, 2.09 Å), PLP_Snyder530 (PDB: 7JIW, 2.30 Å) and XR8-89 (PDB: 7LBR, 2.20 Å) were selected based on the structure’s quality and existing ligand. The selected PDB protein structures were prepared by adding hydrogen atoms and fixing missing side chains using the Protein Preparation Wizard (PrepWiz)^35^, implemented in the Small Discovery Molecule Drug Discovery Suite 2019v.3 (Schrödinger LLC, New York, NY, USA). Sulphate ions and other co-crystallization molecules, such as glycerol (GOL) were removed. Within the catalytic site of PL^pro^ is formed by the catalytic triad, Cys111, His272, and Asp286, sits in a solvent-exposed cleft at the interface of the Thumb and Palm domains^16^. We used the protonation states of the Cys-His-Asp triad reported by Henderson et al.^36^.

Compound 7 (CPD7) proposed binding mode was generated using induced-fit docking^37^, as the crystal structure from the pre-print was not yet available in the PDB database. We employed a cubic grid box of size 12 Å was centralized in the geometric centre of the co-crystallized ligand of 7jir structure. In order to reproduce the published proposed binding mode^24^, restrictions enforcing hydrogen bond interactions with Gly163 and Gly271 were placed. The highest scoring poses reproduced most of the proposed interactions with exception of the hydrogen bond with the Trp93’s side-chain, which suggested our docking to be a good initial pose for simulations.

### Molecular dynamics simulations

Prepared SARS-CoV-2 PL^pro^ structures were simulated as apostructures and in the presence of the ligands, namely GRL-0617, PLP_Snyder530, XR8-89 and CPD7, both non-covalent- and covalently bound to PL^pro^ catalytic cysteine). Molecular Dynamics (MD) simulations were carried out using the Desmond engine^38^ with the OPLS3e force-field^39^ according to a previously described protocol^10^. OPLS3e accomplishes this by incorporating a broad range of chemical moieties with greater and combining them on the fly to generate parameterization, followed by the assignment of partial charges^40^. In short, the system encompassed the protein–ligand/cofactor complex, a predefined water model (TIP3P^41^) as a solvent and counterions (Na^+^ or Cl^-^ adjusted to neutralize the overall system charge). The entire system was treated in a cubic box with periodic boundary conditions (PBC), specifying the shape and the size of the box as 13 Å distance from the box edges to any atom of the protein. Short-range coulombic interactions were calculated using 1 fs time steps and 9.0 Å cut-off value, whereas long-range coulombic interactions were estimated using the Smooth Particle Mesh Ewald (PME) method^42^. Each system was subjected to at least 10 μs simulations (with at least five replicas).

Root mean square deviation (RMSD) values of the protein backbone were used to monitor simulation equilibration and protein folding changes (Supplementary Fig. S4A,C,E,G,H,I). The fluctuation (RMSF) by residues was calculated using the initial MD frame as a reference and compared between ligand-bound and apostructure simulations (Supplementary Fig. S4B,D,F,G,J). The datasets generated and/or analysed during the current study are available in the Zenodo repository, (10.***5281/zenodo.5863718). Data available includes the trajectory raw data and interaction data. MD trajectories were visualized, and figures were generated using PyMOL v.2.5 (Schrödinger LCC, New York, NY, USA).

### Molecular dynamics trajectory analyses

#### Protein–ligand interactions

Atomic interactions and distances were determined using the Simulation Event Analysis pipeline as implemented in Maestro 2019v.4 (Schrödinger LCC). The criteria for protein–ligand H-bond are 2.5 Å distance between the donor and acceptor atoms (D—H···A); ≥ 120° angle between the donor-hydrogen-acceptor atoms (D—H···A); and ≥ 90° angle between the hydrogen-acceptor-bonded atoms (H···A—X). Corresponding requirements for protein-water and water-ligand H-bonds are 2.8 Å (D—H···A); ≥ 110° (D—H···A); and ≥ 90° (H···A—X). Non-specific hydrophobic interactions are defined by the presence of a hydrophobic side chain within 3.6 Å of the ligand's aromatic or aliphatic carbons. Π–π interactions are recorded when two aromatic groups are stacked face-to-face or face-to-edge and within 4.5 Å of distance^43^.

#### Principal component analyses

Principal Component Analysis (PCA) was used to study the main features of PL^pro^ backbone movements. The PCA was conducted for all backbone atoms, which were selected and aligned using scripts (trj_selection_dl.py and trj_align.py) from Schrodinger package 2021v.4. Individual simulations from all runs were merged using the trj_merge.py script into a final trajectory and CMS file. Then, Desmond trajectories were aligned and transformed to xtc-format, keeping only backbone atoms, which were further used to generate the principal components. PCA was conducted for the backbone atoms using GROMACS tools (version 2019) (gmx covar and gmx anaeig)^44^.

For GROMACS analysis. Figures representing the extreme motions were generated and visualized using the PyMOL script Modevectors (https://github.com/Pymol-Scripts/Pymol-script-repo/blob/master/modevectors.py). For further analysis we included the PCs that displayed > 20% individual contribution: PC1 30.8%and PC2 20% (all combined 51.5%). PCA graphics for the 2D projections were generated using a python script, available in the GitHub repository (code: https://github.com/gmf12/plpronalysis). All commands were generated using JuPyter (Matplotlib, Seaborn, Numpy, and Pandas).

#### Domain movement analyses

The distances analysis was used to study loop movements. The distance of selected regions was calculated by the centre of mass distance using the script (trj_asl_distance.py) available on Schrodinger package 2021v.4. The free energy of binding was calculated based on a previous study^45^. The residues to the centre of mass analysis were selected by Ubl (1–60), Thumb (61–176), Finger (177–238), Palm (239–315) and Catalytic triad (Cys111).

#### Dihedral angle analyses

Dihedral angles can help understand the interpretation between the states, because internal coordinates provide the knowledge about the overall motion, undergoing large structural rearrangements^46^. The space of dihedral angles {φ*n*} to the metric coordinate space was employed by equation {*xn* = cos φ*n*, *yn* = sin φ*n*}^46^.

Hence, we selected all the entries for which you want to calculate the torsion and selected the four atoms for the torsion to Tyr268 and Gln269. Dihedral angle analysis and graphics were generated using a python script, available in the GitHub repository (code: https://github.com/gmf12/plpronalysis). All commands were generated using JuPyter (Matplotlib, Seaborn, Numpy, and Pandas).

#### Markov state model (MSM) analysis

MSM generation was conducted with PyEMMA 2^47^. Bayesian MSM was shown following the general recommendations^48^. The individual trajectories of complete PL^pro^ systems (Apo and ligands) were used as an input for MSM generation. For featurization, we used the backbone torsions of whole protein domains (Ubl, Thumb, Triad, Palm, Finger, BL2-groove, and loop; 0–310) in the presence of the ligands and Apo. We validated the final model during the decision process by the VAMP-2/ VAMP-2 backbone torsions score^49^, which was 1.92/1.74 for the final model. The dimensional reduction was conducted with time-lagged independent component analysis (TICA)^50^. The length of lag time τ = 10^3^ ns and 305 dimensions were selected, where the implied timescales were converged. Discretization of the data to microstates was done by k-means clustering (√N used for the number of clusters). Finally, a spectral clustering using the Perron-cluster cluster analysis (PCCA^++^)^51^ assigned the microstates to macrostates. Transition-path theory (TPT) was applied to investigate state transitions and the fux between metastable states^52,53^. MSM graphics were generated using the python script, available in the GitHub repository (code: https://github.com/gmf12/plpronalysis). The validation of MSM models were shown in Supplementary Fig. S6.

## Supplementary Information

Supplementary Information.

## Data Availability

The datasets generated and/or analysed during the current study are available in the Zenodo repository, (10***.5281/zenodo.5863718). Data available includes the trajectory raw data and interaction data. Dihedral angle and MSM models and analysis were generated using a python script, available in the GitHub repository (code: https://github.com/gmf12/plpronalysis).
