# Supplementary material for: Inhibitor induced conformational changes in SARS-COV-2 papain-like protease
Source: Sci Rep. 2022 Jul 8;12:11585. doi: 10.1038/s41598-022-15181-y (PMC9270405; doi:10.1038/s41598-022-15181-y)
Supplement: Supplementary file 1 — Supplementary Information. [file 41598_2022_15181_MOESM1_ESM.docx]

**Supplementary Figures and Tables**

*Inhibitor induced conformational changes in SARS-COV-2 papain-like protease*

**Glaucio Monteiro Ferreira^1^, Thanigaimalai Pillaiyar ^2^, Mario Hiroyuki Hirata1, Antti Poso^3,4^ and Thales Kronenberger^2,3,4,5*^**

**Corresponding authors:*

*Tel:* [*kronenberger7@gmail.com*](mailto:kronenberger7@gmail.com) *(T.K.), +49 (0)7071-29 74575*

Table S1. Y83 frequency of interaction.

| **Y83 interactions %** | | | | | | |
| --- | --- | --- | --- | --- | --- | --- |
| **Residue** | **Apo** | **GRL-0617** | **PLP_Snyder530** | **XR8-89** | **Cpd7 (non-covalent)** | **Cpd7 (covalent)** |
| Y72 | 45 | 40 | 35 | 34 | 41 | 44 |
| N146 | 66 | 65 | 63 | 55 | 67 | 67 |

Table S2. Ligand frequency of interaction.

| **Hydrogen bond interactions %** | | | | | |
| --- | --- | --- | --- | --- | --- |
| **Residue** | **GRL-0617** | **PLP_Snyder530** | **XR8-89** | **Cpd7 (non-covalent)** | **Cpd7 (covalent)** |
| G163 |  |  |  | 75 | 94 |
| R166 | 21.3 |  |  |  |  |
| G266 | 55.82 |  | 77.7 |  |  |
| Y268 | 61.82 | 82.7 | 55.78 | 53 | 70 |
| Q269 | 76.7 | 64.75 | 52.55 | 74 | 96 |
| G271 |  |  |  | 57 | 41 |
|  |  |  |  |  |  |
| **Hydrophobic interactions %** | | | | | |
| **Residue** | **GRL-0617** | **PLP_Snyder530** | **XR8-89** | **Cpd7 (non-covalent)** | **Compound 7 (covalent)** |
| P248 | 30.45 | 29.67 | 48.98 | 51 | 65 |
|  |  |  |  |  |  |
| **π-π interactions %** | | | | | |
| **Residue** | **GRL-0617** | **PLP_Snyder530** | **XR8-89** | **Cpd7 (non-covalent)** | **Cpd7 (covalent)** |
| Y268 | 92.56 | 90.61 | 93.77 | 91 | 96 |

Table S3. D164 frequency of interaction.

| **D164 interactions %** | | | | | | |
| --- | --- | --- | --- | --- | --- | --- |
| **Residue** | **Apo** | **GRL-0617** | **PLP_Snyder530** | **XR8-89** | **Cpd7 (non-covalent)** | **Cpd7 (covalent)** |
| R166 | 42 | 22 | 44 | 56 | 45 | 58 |
| E167 | 62 | 66 | 64 | 67 | 67 | 79 |
| T168 | 91 | 95 | 91 | 96 | 93 | 94 |


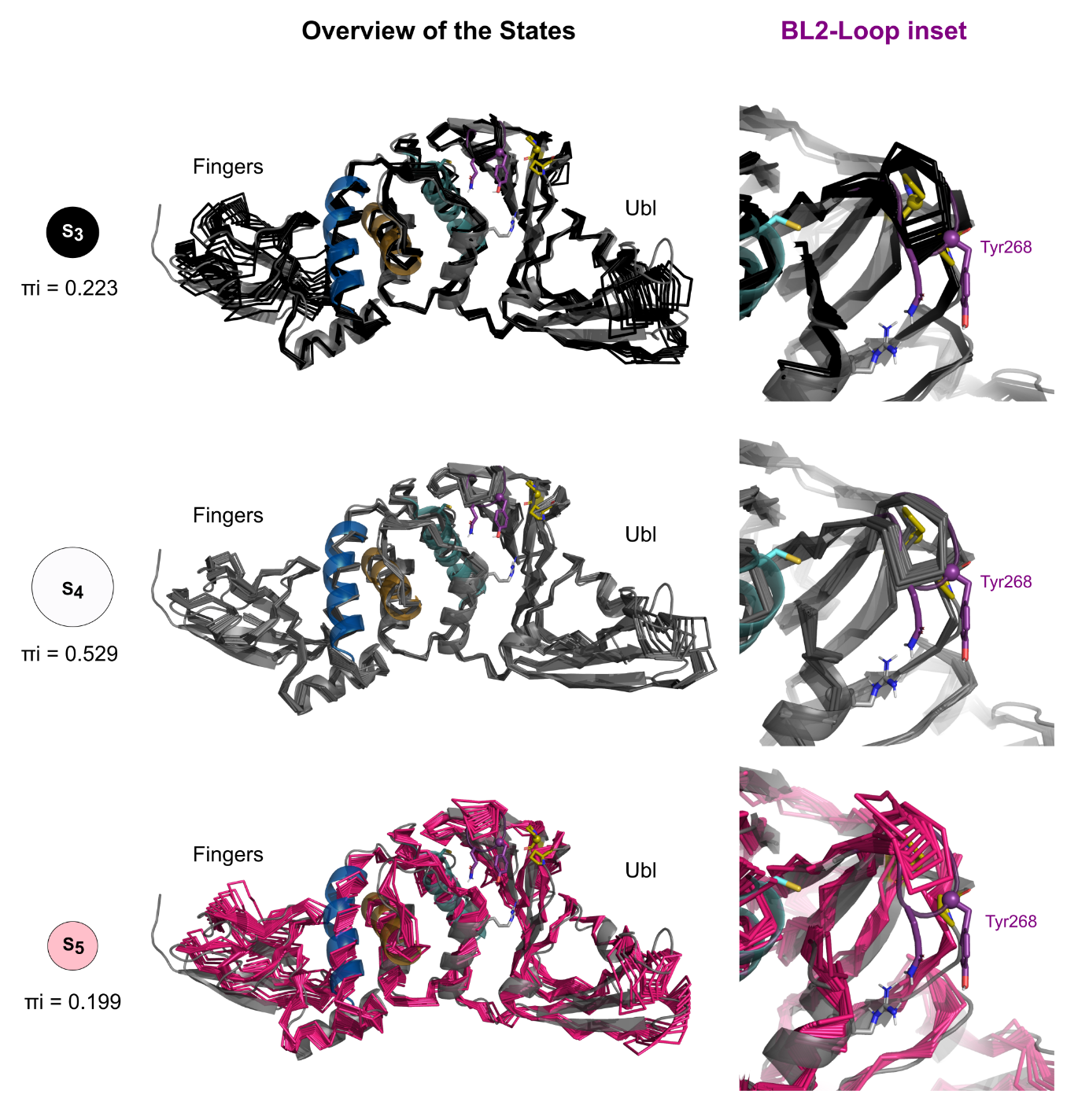


**Supplementary Figure S1.** The three highest ranked metastable states, according to their probability, an overview (left) and a inset zoom of the BL2 loop region (right). Each metastable state (S) is illustrated with ten representative structures (coloured ribbons), superimposed to a transparent cartoon with the original crystal structure. Equilibrium probability (πi) of distributions for each state is indicated below the conformations together with circles with an area representing the changes induced by the systems, values near to 1.0 more probable than values close to 0.


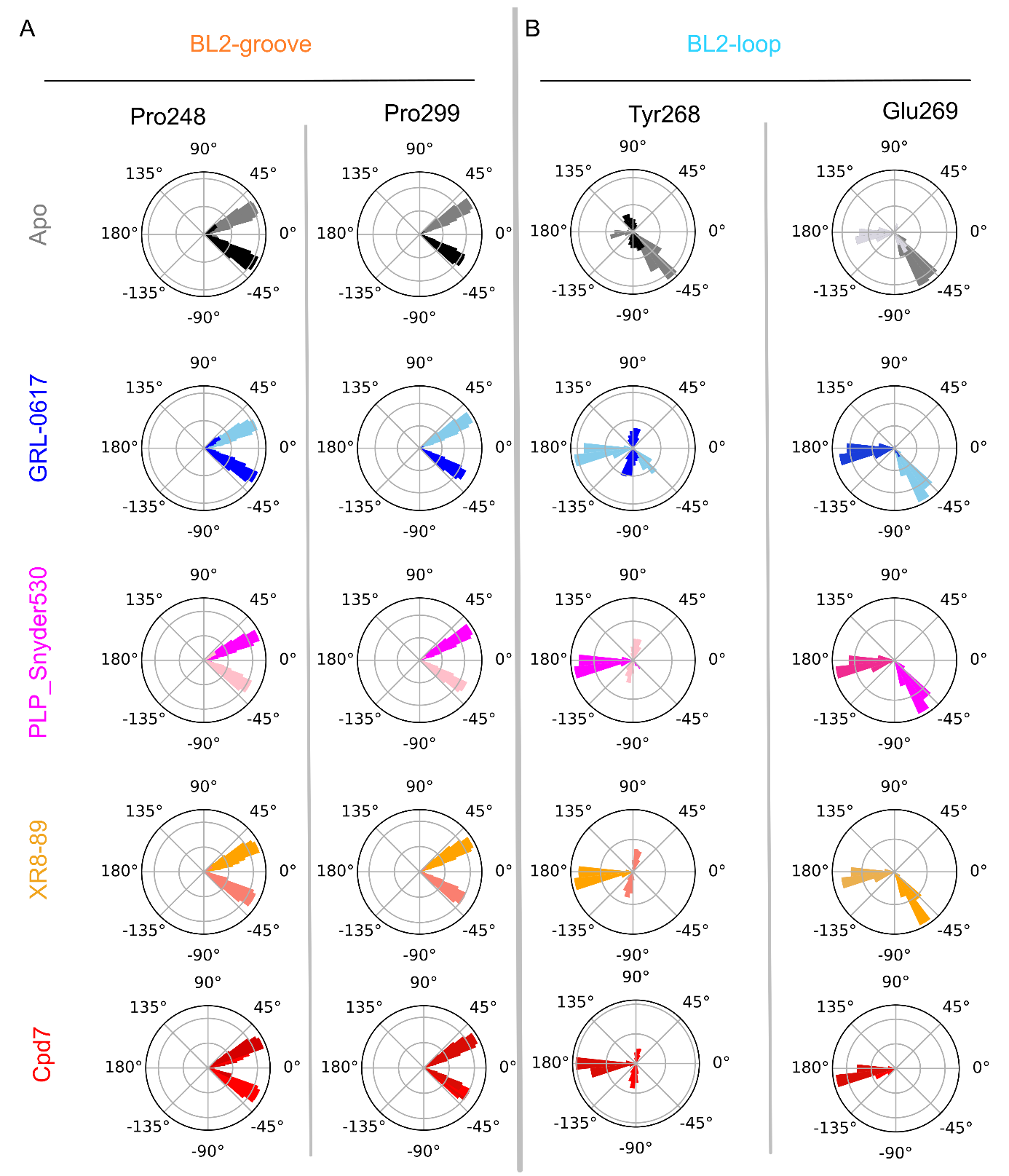


**Supplementary Figure S2.** Dihedral angle distribution analysis for the residues BL2-groove (A) and BL2 loop region (B). Graphics are coloured according to: Apostructure by grey, GRL-0617 sky-blue; PLP_Snyder530, magenta, XR8-89, orange and Cpd7, red.


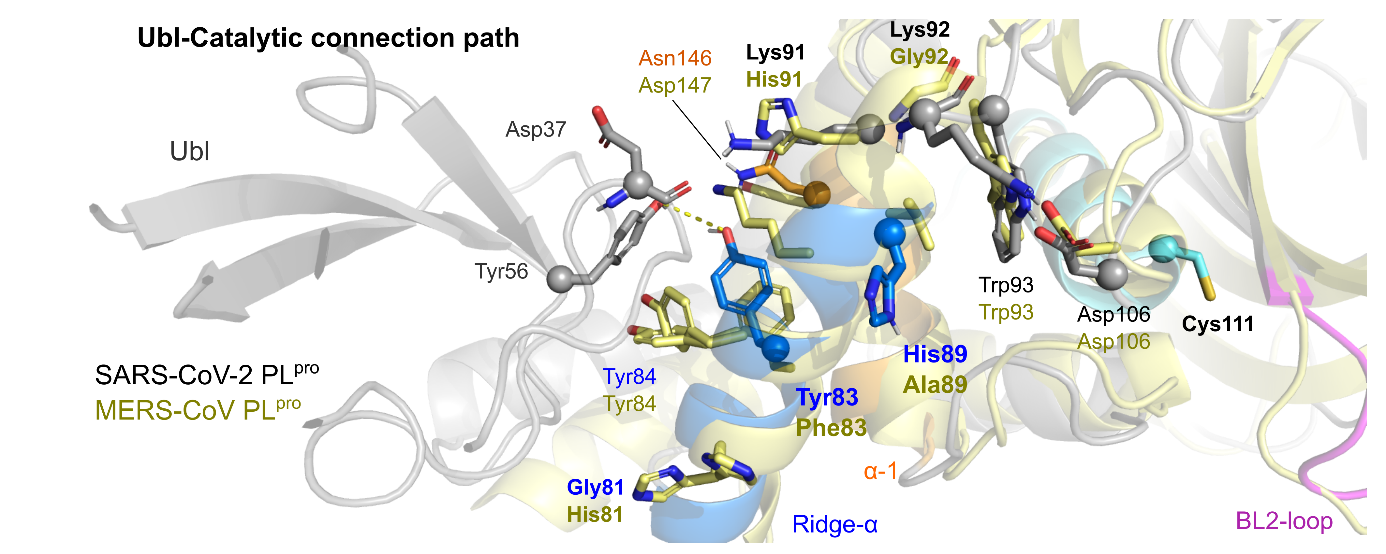


**Supplementary Figure S3.** Comparison between MERS-CoV and SARS-CoV-2 PL^pro^ structures, relevant residue changes and conserved site.


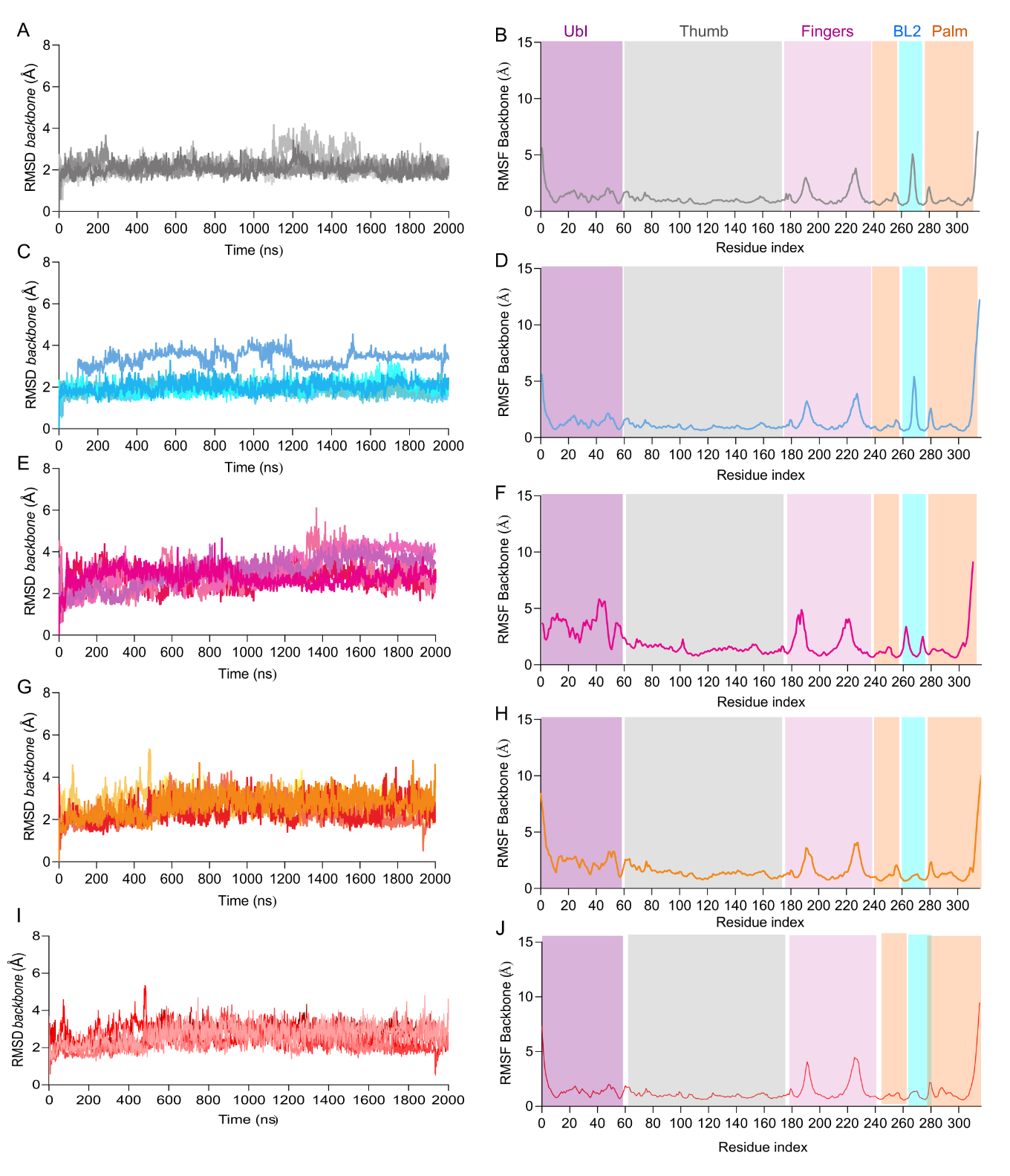


**Supplementary Figure S4.** RMSD and RMSF analysis of the SARS-CoV-2 PL^pro^ (apostructure and with ligands GRL-0617, PLP_Snyder530 and XR8-89), each replica (#1, #2 and #3 are represented by individual colours). (**A**) RMSD of the apostructure from PDB:7lbr; (**B**) RMSF of the apo structure from PDB:7lbr; (**C**) RMSD (GRL-0617) from PDB:7jir; (**D**) RMSF (GRL-0617) from PDB:7jir; (**E**) RMSD (PLP_Snyder530) from PDB:7jiw, (**F**) RMSF (PLP_Snyder530) from PDB:7jiw, (**G**) RMSD (XR8-89) from PDB:7lbr, (**H**) RMSF (XR8-89) from PDB:7lbr and (**I**) RMSD (Cpd7) and (**J**) RMSF (Cpd7) Sanders et all 2022. Average and standard deviation values are shown as solid and dashed lines, respectively, from five replicas. RMSD: Root-mean-square deviation of atomic positions; RMSF: Root-mean-square deviation of atomic positions.


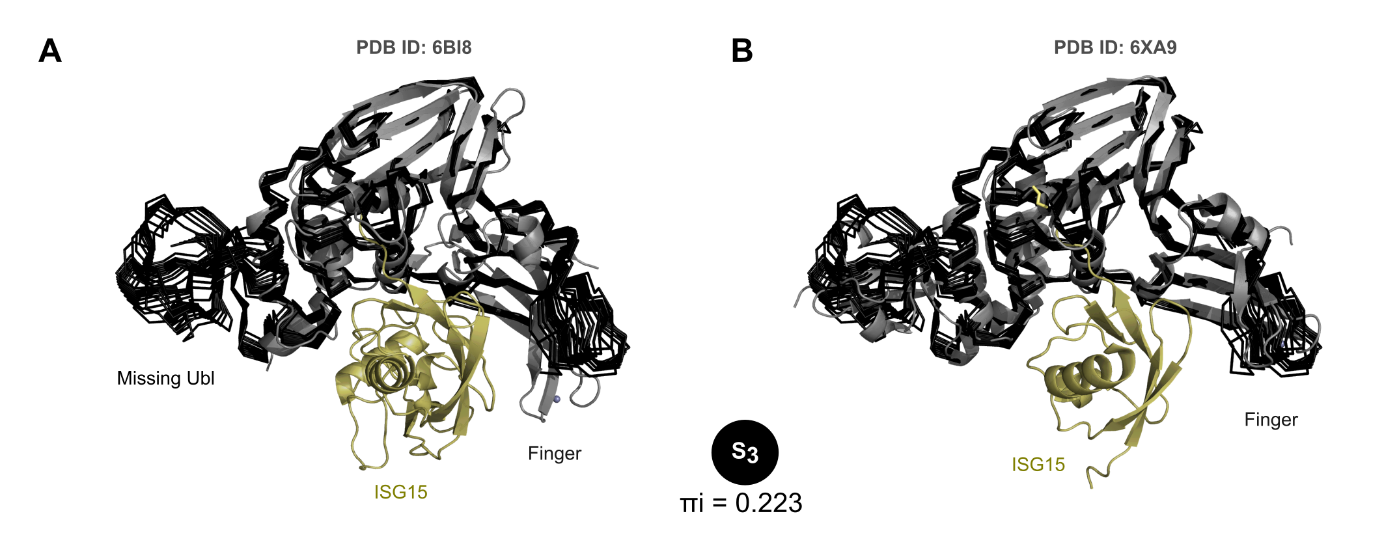


**Supplementary Figure S5.** Substrate bound PL^pro^ structures superimposed with representative frames of the metastable S3 (as an example for Finger flexible state). A) PDB ID 6BI8 (a mutant lacking the Ubl domain) and B) PDB 6XA9 (complete sequence)


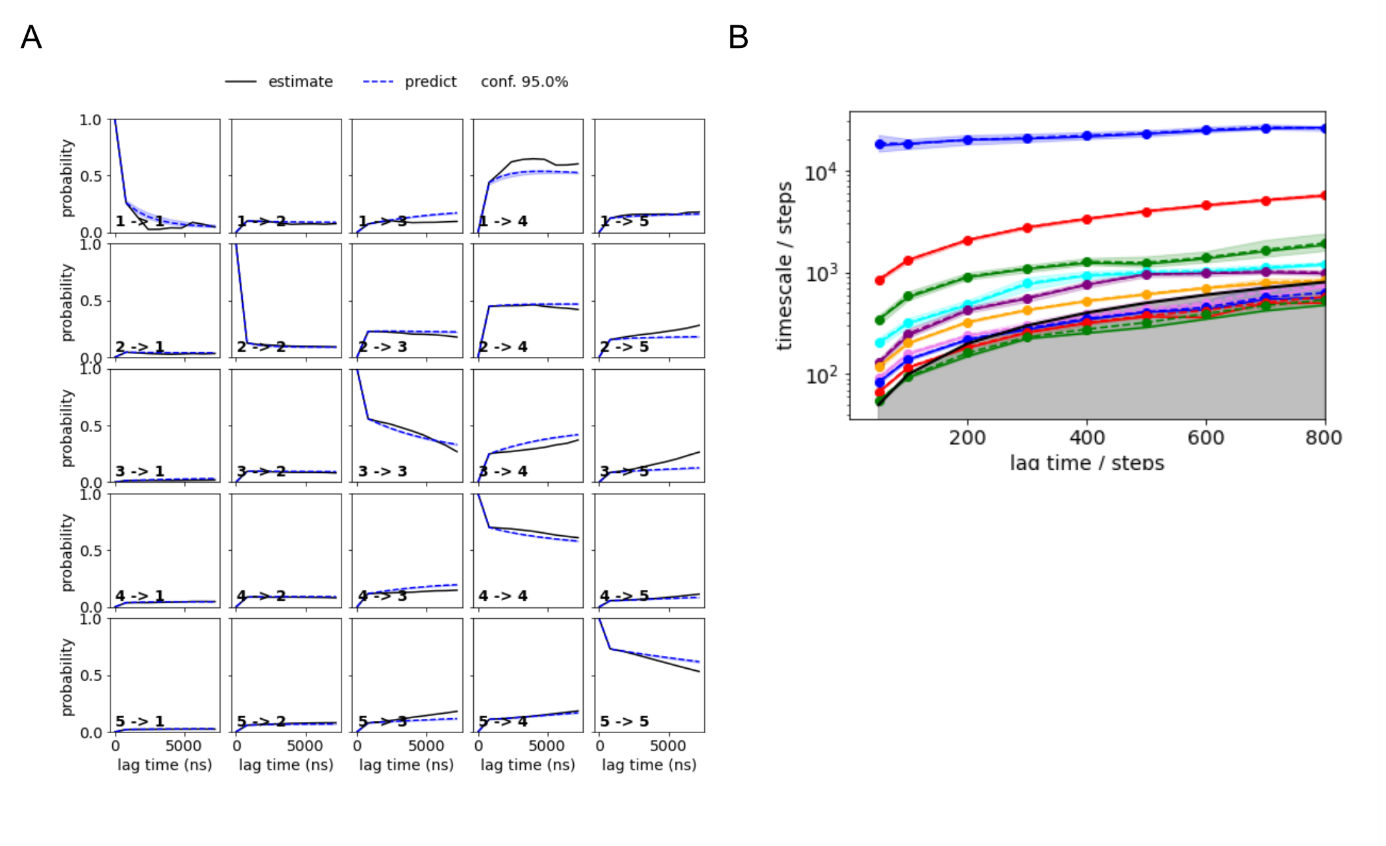


**Supplementary Figure S6.** Validation of Markov State Models. Chapman-Kolmogorov tests demonstrate that the models follow expected estimates (A). In the selected lag times the implied timescales are converged (B).
